# Prenatal diagnosis and ultrasonographic findings of partial trisomy of chromosome 6q: A case report and review of the literature

**DOI:** 10.1097/MD.0000000000024091

**Published:** 2021-01-15

**Authors:** Linlin Li, Yang Yu, Han Zhang, Yuting Jiang, Ruizhi Liu, Hongguo Zhang

**Affiliations:** Center for Reproductive Medicine and Center for Prenatal Diagnosis, First Hospital, Jilin University, Changchun, China.

**Keywords:** familial translocation, partial trisomy of 6q, prenatal diagnosis, ultrasonographic findings

## Abstract

**Rationale:**

Partial trisomy of the long arm of chromosome 6 syndrome is a rare chromosomal disorder with distinctive phenotypic expressivity, in which cytogenetic abnormalities are usually reported in infancy and childhood. Ultrasonographic findings on trisomy of the distal long arm of chromosome 6 in previous studies are limited.

**Patient concerns:**

A 32-year-old, gravida 6, para 1, pregnant woman who had 4 spontaneous abortions underwent a clinical ultrasound examination at 26 weeks of gestation.

**Diagnoses:**

Ultrasonographic findings were microcephaly, an acoustic image of a transparent septum, a flat nasal bridge, right pulmonary artery stenosis, and a single umbilical artery. Cytogenetic and single-nucleotide polymorphism array analyses were performed to estimate genetic factors of this diagnosis by amniocentesis.

**Interventions:**

After genetic counseling, the patient and her husband opted to terminate the pregnancy.

**Outcomes:**

Cytogenetic examination of the fetus showed the karyotype 46,XX,der(20)t(6;20)(q24;p13). The single-nucleotide polymorphism (SNP) array showed a 22.104-Mb duplication of 6q24.3q27 and a 0.784-Mb deletion of 20p13.

**Lessons:**

Ultrasonographic findings of fetal abnormalities, including microcephaly, an acoustic image of a transparent septum, a flat nasal bridge, right pulmonary artery stenosis, and a single umbilical artery, may be related to a 22.104-Mb duplication of 6q24.3q27 and a 0.784-Mb deletion of 20p13. More ultrasonographic and genotype studies are required to extend the phenotypic characterization of partial trisomy 6q syndrome.

## 1 Introduction

Partial trisomy of the long arm of chromosome 6 syndrome is a rare chromosomal disorder with distinctive phenotypic expressivity, such as severe physical and mental retardation, hypertelorism, feeding difficulties, and dysmorphic features (microcephaly, prominent forehead, flat nasal bridge, downward slanting palpebral fissures, carp mouth, micrognathia, short webbed neck, club feet, and flexion deformity).^[1–3]^ Cytogenetic abnormalities are usually reported in infancy and childhood.^[4]^ Most patients who carry unbalanced translocations have partial trisomy 6q and partial monosomy of another chromosome.^[5]^ We report a case of partial trisomy 6q and partial monosomy 20p due to familial translocation t(6;20)(q24;p13). In our case, multiple anomalies were detected by ultrasound, and karyotype was ascertained by prenatal cytogenetic diagnosis in amniocytes. We also briefly review the literature.

## 2 Methods

The study protocol was approved by the Ethics Committee of the First Hospital, Jilin University (No. 2020-249). The patient and her husband signed a written informed consent for publication of the case.

### 2.1 Cytogenetic analysis

Amniotic fluid cells were obtained by amniocentesis at 24 weeks of gestation. Amniocytes were collected after centrifugation at 252× *g* for 6 minutes. These cells were then inoculated in 25-cm^2^ flasks with 5 mL of culture medium and cultured in carbon dioxide incubators for 10 days. G-banding for chromosomal sample preparation was performed by standard methods. In this case, 20 metaphase cells were counted and 6 metaphase cells were analyzed. Chromosomal abnormality was described according to the International System for Human Cytogenetic Nomenclature 2013.^[6]^

### 2.2 Single-nucleotide polymorphism array analysis

Genomic DeoxyriboNucleic Acid (DNA) was extracted from 10 mL of uncultured amino fluid cells using a QIAamp DNA MINI kit (Qiagen, Hilden, Germany). Single-nucleotide polymorphism (SNP) array analysis was performed using the Human CytoScan 750K BeadChip (Affymetrix, San Diego, CA) and the data of these images were analyzed with Chromosome Analysis Suite v3.3 software. The final results were analyzed by the Database of Chromosomal Imbalance and Phenotype in Humans using Ensembl Resources, Online Mendelian Inheritance in Man, the database of genomic variants, and National Center for Biotechnology Information.

## 3 Case presentation

**Figure 1:**
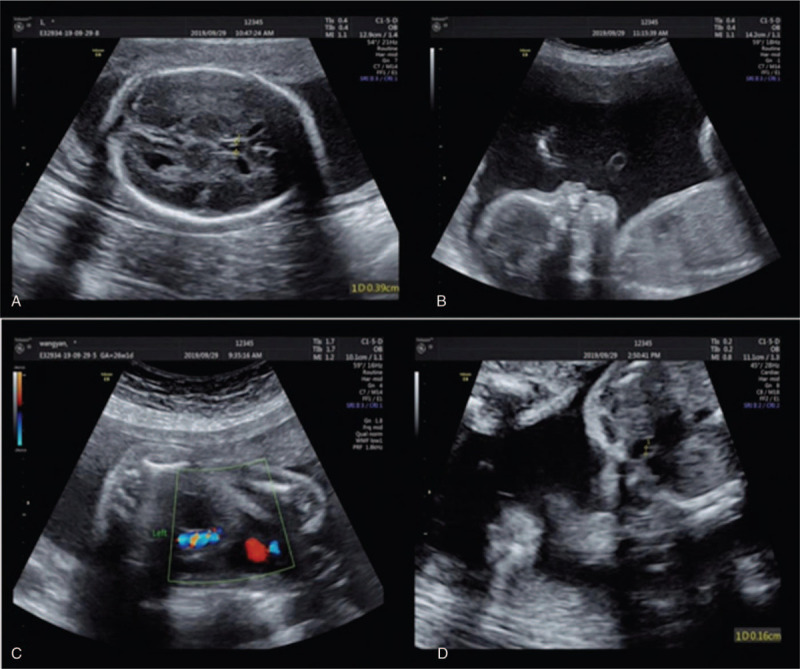
Prenatal ultrasound at 20 wk of gestation shows the fetus with an acoustic image of a transparent septum (A), absence or hypoplasia of the nasal bone (B), a single umbilical artery (C), and right pulmonary artery stenosis (D).

A 32-year-old, gravida 6, para 1, pregnant woman who had 4 spontaneous abortions underwent a clinical ultrasound examination at 26 weeks of gestation. Ultrasonographic findings showed abnormality of the biparietal diameter (5.3 cm), which indicated that the gestational age was <24 weeks (microcephaly), an acoustic image of a transparent septum, absence or hypoplasia of the nasal bone (flat nasal bridge), right pulmonary artery stenosis, and a single umbilical artery (Fig. 1). Subsequently, the pregnant woman underwent cytogenetic and SNP array analysis at 26.5 weeks of gestation because of the abnormal ultrasound indicators.

**Figure 2:**
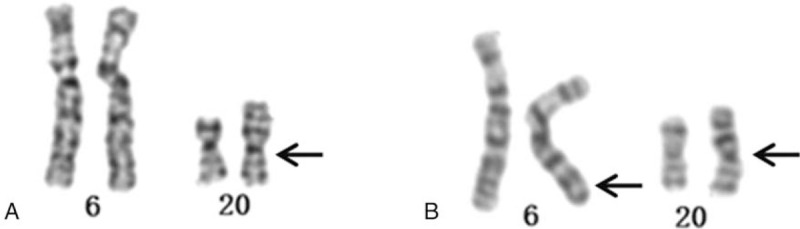
G-banded chromosomes 6 and 20 from the fetus (A) and the father (B).

**Figure 3:**
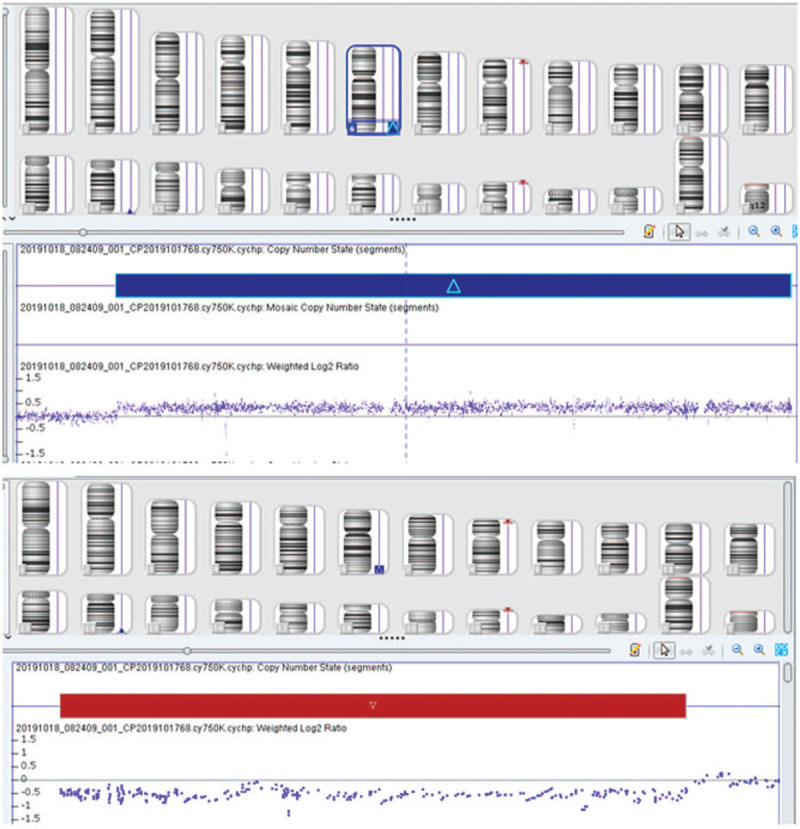
Single-nucleotide polymorphism array on uncultured amniocytes shows 6q24.3q27 duplication and 20p13 deletion.

A cytogenetic examination of the fetus showed the karyotype 46,XX,der(20)t(6;20)(q24;p13) (Fig. 2). The SNP array showed a 22.104-Mb duplication of 6q24.3q27 and a 0.784-Mb deletion of 20p13 (6q24.3q27 (148810213–170914297) × 3, 20p13(61661–845821) × 1) (Fig. 3). The couple underwent cytogenetic detection. The father's karyotype showed a balanced reciprocal translocation of 46,XY,t(6;20)(q24;p13) (Fig. 2). The mother's karyotype was normal.

The pregnant woman and her husband were nonconsanguineous and healthy, and their first child was a healthy boy. The mother denied any exposure to alcohol, smoking, teratogenic agents, infectious diseases, or irradiation during this pregnancy. Because of an unbalanced segregation of paternal t(6;20), the fetus had 2 normal chromosomes 6, 1 normal chromosome 20, and 1 derivative chromosome 20, leading to duplication of chromosome 6q24 → qter, and a deficiency of chromosome 20p13 → pter. After genetic counseling, the couple opted to terminate the pregnancy.

## 4 Discussion

The first report of partial trisomy of the distal long arm of chromosome 6 was described by de Grouchy et al in 1969.^[7]^ Since this time, other additional cases have been reported. Owing to common clinical manifestations with a similar duplication, partial trisomy of the long arm of chromosome 6 has been established as a clinically diagnosable syndrome. Most of the reported cases of this condition were diagnosed at birth or in childhood. The chief clinical features of partial trisomy of the distal long arm of chromosome 6 approximately resemble due to duplication of the same chromosome. According to previous studies, although fragments of the duplication are unequal in size, partial trisomy of the distal long arm of chromosome 6 (6q26 → qter) produces typical clinical manifestations. Additional genetic material from the long arm of chromosome 6 does not consistently alter the clinical phenotype.^[8,9]^ In some cases, there are other abnormal phenotypes. These are different from the characteristic phenotype owing to the varying length of the long arm duplication of chromosome 6 and deleted segments of another chromosome involved in translocation.^[8]^

Previous studies have shown that only 4 cases of partial trisomy of the distal long arm of chromosome 6 were diagnosed during pregnancy. Three of these four cases showed an abnormal phenotype by ultrasonography. Another case had amniocentesis performed at 17 weeks of gestation because of spontaneous abortion of partial trisomy 6q at the gestational week of 4 months.^[10–12]^ The abnormal phenotype in the present case was detected by ultrasonography at 26 weeks of gestation, and then cytogenetic and SNP array analyses were performed by amniocentesis to diagnose the syndrome. This is the first case of diagnosis of partial trisomy of the distal long arm of chromosome 6 by using SNP array with cytogenetic analysis. We identified the breakpoints at 6q24 and 20p13. The present case was a female fetus with a 22.104-Mb duplication of 6q24 → qter and a 0.784-Mb deletion of 20p13 → pter.

Morphological abnormalities of partial trisomy 6q according to a review of the literature are shown in Table 1. Ultrasound findings in the previous literature showed that 80% cases had the clinical phenotype of microcephaly and a flat nasal bridge, and 50% cases had the phenotype of a cardiovascular anomaly (Table 1). Other typical malformations were not found in ultrasound findings of the present case. Ultrasonographic findings on duplication of the distal long arm of chromosome 6 in previous studies were limited, and they varied between patients.

**Table 1:** Phenotypic characteristics with partial trisomy of the long arm of chromosome 6 in our case and published literature.

	Present case	Miyabara et al^[15]^	Uhrich et al^[10]^	Kulharya et al^[11]^	Valerio et al^[12]^	Pivnick et al^[8]^	Tipton et al^[13]^	Frarchino et al^[9]^	Chase et al^[14]^
Duplication segment	6q24 → ter	6q21 → tter	6q23.3 → ter	6q22.2 → ter	6q21 → q22	6q23 → ter	6q21 → ter	6q26 → ter	6q26 → ter
Presumed deleted segment	20p13 → ter	7p22 → ter	15q26.3 → ter	1p36.3 → ter	NA	15p12 → ter	3p25 → ter	3p23 → ter	3q2402 → ter
Age	26 wk of preg.	19 wk of preg.	35 wk of preg.	23.5 wk of preg.	23 wk of preg.	New born	14 yrs	New born	New born
Sex	F	M	NA	M	F	M	M	M	F
Microcephaly	+	−	+	−	+	+	+	+	+
Flat nasal bridge	+	+	−	+	+	−	+	+	−
Crap mouth	−	+	+	+	+	+	+	+	−
Micrognathia	−	−	−	−	+	+	+	+	+
Short webbed neck	−	+	−	+	+	+	+	+	+
Club foot	−	+	−	+	+	+	+	+	−
Cardiovascular anomaly	+	+	+	+	−	+	−	−	−
Flexion deformity	−	+	+	−	+	+	+	−	+
Rental anomaly	−	+	−	+	−	−	−	−	−

M = male, F = female, NA = not applicable.

The limitation of this study is that we could not provide the results of a fetal autopsy because the mother refused consent for this research.

## 5 Conclusions

Ultrasonographic findings of fetal abnormalities, including microcephaly, an acoustic image of a transparent septum, a flat nasal bridge, right pulmonary artery stenosis, and a single umbilical artery, may be related to a 22.104-Mb duplication of 6q24.3q27 and a 0.784-Mb deletion of 20p13. More ultrasonographic and genotype studies are required to extend the phenotypic characterization of partial trisomy 6q syndrome.

## Acknowledgments

We thank Ellen Knapp, PhD, from Liwen Bianji, Edanz Group China (www.liwenbianji.cn/ac), for editing the English text of a draft of this manuscript.

## Author contributions

**Conceptualization:** Hongguo Zhang.

**Data curation:** Yuting Jiang.

**Funding acquisition:** Ruizhi Liu.

**Investigation:** Linlin Li, Han Zhang.

**Methodology:** Yang Yu, Han Zhang, Yuting Jiang.

**Resources:** Yang Yu.

**Writing – original draft:** Linlin Li.

**Writing – review & editing:** Ruizhi Liu, Hongguo Zhang.

## Abbreviations

Abbreviations: DNA = DeoxyriboNucleic Acid, SNP = single-nucleotide polymorphism.
